# Application of prehabilitation program in elderly patients undergoing esophageal cancer surgery

**DOI:** 10.3389/fonc.2025.1605647

**Published:** 2025-07-11

**Authors:** Kun Zhou, Fengjuan Cai, Xiao Shao, Caifeng Luo, Zihao Liu

**Affiliations:** ^1^ Interventional and Geriatric Department, The Affiliated Suqian Hospital of Xuzhou Medical University, Suqian, Jiangsu, China; ^2^ Endoscopy Center, Jiangsu Province (Suqian) Hospital, Suqian, Jiangsu, China; ^3^ School of Medicine, Jiangsu University, Zhenjiang, Jiangsu, China; ^4^ Oncology Department, The Affiliated Suqian Hospital of Xuzhou Medical University, Suqian, Jiangsu, China

**Keywords:** elderly, esophageal cancer, prehabilitation, surgery, program application

## Abstract

**Objective:**

To explore the effectiveness of prehabilitation program on elderly patients undergoing esophageal cancer surgery.

**Methods:**

A total of 96 elderly esophageal cancer surgery patients from a tertiary hospital were selected. The control group included 48 patients treated from August to November 2023, and the prehabilitation group consisted of 48 patients treated from December 2023 to March 2024. The control group received routine care from the time of diagnosis until surgery, while the prehabilitation group received the prehabilitation program in addition to routine care. The nutritional status, hospital anxiety and depression scale (HADS), six-minute walking distance (6MWD), and quality of life (SF-36) were compared between the two groups at different time points before and after the intervention. Additionally, postoperative hospital stay, time to chest drain removal, time to first ambulation, and complications were evaluated.

**Results:**

During the research process, in the control group, one case fell out, while two cases fell out in the prehabilitation group. The t-test and Mann Whitney U test showed that at different time points after the intervention, the indicators in the prehabilitation group were significantly better than those in the control group (*P*<0.05), although there was no significant difference in complications (*P*>0.05). Repeated measures ANOVA indicated that there were interaction effects, intervention effects, and time effects on HADS, 6MWD, and SF-36 scores at each time point in both groups (*P*<0.05).

**Conclusion:**

Prehabilitation has a positive effect on improving the functional reserve, nutritional status, and psychological well-being of elderly patients undergoing esophageal cancer surgery, thus promoting postoperative recovery.

## 1 Introduction

Esophageal cancer is one of the common malignant tumors. The incidence and mortality of esophageal cancer in my country are much higher than those in other countries (1), and elderly patients account for more than 70% (2), putting greater pressure on society and families. Surgery is the main treatment modality, but there is a “window period” between tumor diagnosis and surgery. During this time, elderly patients face a high risk of malnutrition (3), and are prone to experiencing severe anxiety and depression, with some even developing suicidal tendencies (4). Additionally, their physical endurance declines, leading to reduced surgical tolerance and an increased risk of perioperative mortality (5). Therefore, effectively enhancing the preoperative functional status of elderly patients with esophageal cancer improves their prognosis and quality of life, which requires urgent attention.

In recent years, prehabilitation has provided a new idea to improve the perioperative condition of patients. It is a rehabilitation intervention based on accelerated rehabilitation surgery, which includes a comprehensive program of exercise, nutritional supplementation and anxiety management (6). The implementation of prehabilitation has significantly improved the nutritional and functional status, as well as the prognosis, of surgical patients. However, both domestic and international prehabilitation management lacks standardization, with low implementation rates, and it is often initiated only after hospitalization. There is also a lack of studies exploring prehabilitation practices from the time of diagnosis to the preoperative period, particularly for elderly esophageal cancer patients (7). Therefore, this study preliminarily applied a prehabilitation program, constructed based on a review of the literature and expert consultation, to elderly esophageal cancer patients immediately after diagnosis.

## 2 Materials and methods

### 2.1 Study subjects

The study included 96 elderly patients who underwent esophageal cancer surgery at a tertiary hospital. Using the first day of outpatient pathological diagnosis as the baseline, the patients were divided into two groups: 48 patients admitted between August 2023 and November 2023 were assigned to the control group, while 48 patients admitted between December 2023 and March 2024 were assigned to the prehabilitation group. All participants voluntarily took part in the study and signed the “Informed Consent Form”. The study was approved by the Ethics Committee of the Medical School of Jiangsu University (NO.20221019-5).

### 2.2 Inclusion, exclusion, and withdrawal criteria

#### 2.2.1 Inclusion criteria

① Patients aged ≥60 years with mobility; ② Preoperatively diagnosed with esophageal cancer and eligible for surgery; ③ No preoperative special biological therapy; ④ Classified as American Society of Anesthesiologists (ASA) grade 1-2.

#### 2.2.2 Exclusion criteria

① Patients with comorbid conditions such as psychiatric disorders, other malignancies, consciousness disorders, severe bronchial asthma or emphysema, severe heart failure, etc.; ② Participation in other clinical intervention studies.

#### 2.2.3 Withdrawal criteria

① Voluntary withdrawal during the treatment period; ② Discontinuation of treatment or death.

### 2.3 Intervention methods

#### 2.3.1 Intervention methods for the control group

After diagnosis, routine preoperative care is provided as follows (1): Preoperative guidance: ① Distribute health guidance manuals to familiarize patients with the basic medical process, educate them on disease-related knowledge, and encourage smoking and alcohol cessation; ② Advise patients with abnormal blood glucose or blood pressure to take their medications on time; ③ Explain the anesthesia method and fasting (food and drink) times to the patient; ④ Instruct patients on ankle pump exercises and effective coughing techniques (2). Nutritional care: Provide individualized nutritional care based on the patient’s condition and tolerance, and supplement nutritional formulations as prescribed (3). Psychological care: actively communicate with patients to alleviate concerns about the surgery (4). Pain management: Assess the patient’s pain in a timely manner and administer analgesics as needed under medical guidance.

#### 2.3.2 Intervention methods for the prehabilitation group

In addition to routine care, a prehabilitation protocol is implemented from the first day of diagnosis until the day before surgery, as follows:

##### 2.3.2.1 Formation of the prehabilitation intervention team

A multidisciplinary prehabilitation team is formed, consisting of the head nurse from thoracic surgery, doctors, nurses, anesthetists, nutritionists, rehabilitation therapists, psychologists, counselors, and master’s students. The qualifications, roles, and responsibilities of the prehabilitation team members are clearly defined:① The chief nurse is responsible for developing the project plan, team training, and quality control; ② The thoracic surgeon is in charge of diagnosing and treating the disease; ③ The rehabilitation therapist and anesthetist formulate personalized exercise rehabilitation plans; ④ The nutritionist assesses the patient’s nutritional status, determines their nutritional and energy requirements, and develops a corresponding nutritional prescription; ⑤ The psychologist and counselor are responsible for psychological counseling and support; ⑥ Nurses provide guidance on diet, exercise, and psychological health, implement the plan, and assess the patient’s adherence and the effectiveness of the intervention; ⑦ The Master of nursing is responsible for collecting, organizing, and analyzing the data.

##### 2.3.2.2 Development and implementation of the prehabilitation program

After conducting a comprehensive literature search and screening, a total of 14 studies were included, consisting of 3 guidelines (8–10), 1 expert consensus (11), 1 systematic review (12), 5 randomized controlled trials (RCTs) (13–17), and 4 quasi-experimental studies (18–21). Evidence was extracted, and based on the physiological and psychological frailties of elderly patients with esophageal cancer, an initial draft of the program was developed. Sixteen experts were invited to participate in two rounds of consultation. All experts met the following criteria: ① they were from tertiary general hospitals; ② they had been working in relevant fields for over 10 years; ③ they held senior or higher professional titles. The expert authority coefficient was 0.897, with a positive coefficient of 100%. The variation coefficient for each item was ≤0.19, and Kendall’s coefficient ranged from 0.213 to 0.384, indicating a high level of agreement and coordination among the experts, ensuring the reliability of the prehabilitation program. The specific program is detailed in **Table 1**.

**Table 1:** Prehabilitation protocol for elderly esophageal cancer surgery patients.

Intervention	Specific measures
Assessment and education	① At the time of enrollment, the intervention team will conduct nutritional assessments using the BMI, NRS2002 scale, nutritional evaluation tools (PG-SGA scale), and laboratory indicators such as albumin. Psychological assessment will be conducted using the HADS, and exercise risk assessment will be carried out using the 6MWT and pulmonary function tests. Based on these assessments, the patient’s prehabilitation risk will be comprehensively determined. Patients with low risk (BMI ≥ 17.5 kg/m², NRS2002 score < 3, 6MWD ≥ 400 m, no pulmonary dysfunction, and no signs of anxiety or depression) will undergo home-based prehabilitation before hospitalization, while medium- and high-risk patients will regularly visit the hospital for prehabilitation guidance. ②A prehabilitation manual for esophageal surgery will be provided, with explanations and instructions given. ③ WeChat group: Patients or their family members will join a specialized prehabilitation WeChat group, where rehabilitation education materials, both written and video, covering the home-based phase, hospital admission, preoperative, and postoperative phases will be shared. The group will guide patients in completing prehabilitation exercises, and patients will check in and provide feedback on any issues. ④ Smoking cessation: Patients are encouraged to quit smoking as early as possible, and appropriate medications and support will be provided as per medical advice.
Nutritional optimization	① For patients with malnutrition, interventions should follow the five-step principle based on individualized conditions while controlling symptoms: a. Diet plus nutritional education: As the foundation; b. Diet plus oral nutritional supplementation: To compensate for inadequate dietary intake; c. Full enteral nutrition: For patients unable to eat or meet nutritional requirements; d. Partial enteral nutrition combined with partial parenteral nutrition: For those completely unable to eat; e. Total parenteral nutrition: For patients with digestive tract obstruction, etc. ② High-quality protein supplementation: Daily protein intake should be 1.2-1.5g/kg. To compensate for insufficient dietary intake, patients should consume whey protein within one hour after exercise. ③ Select the appropriate type and route of nutrition (oral food or nutritional supplements, nasogastric enteral nutrition preparations, intravenous parenteral nutrition preparations) based on the patient’s condition, and begin supplementation immediately after diagnosis, continuing daily until the day before surgery. ④ Correction of anemia: For iron-deficiency anemia, patients should take iron supplements as prescribed; for megaloblastic anemia, oral vitamin B12 or folic acid is recommended. ⑤ Preoperative fluid replenishment on the day of surgery: Administer 500ml of 5% glucose normal saline (GNS) intravenously to improve metabolism.
Psychological care	① Encourage patients to express their feelings, listen patiently, and provide emotional reassurance, using body language such as holding hands or patting shoulders to comfort them. ② Encourage patients to practice self-regulation techniques, such as listening to soft music before bed, doing home relaxation exercises, or meditating. ③ If the Hospital Anxiety and Depression Scale (HADS) score indicates a risk of anxiety or depression, professional intervention by a psychologist or counselor should be provided, with targeted psychological guidance and rehabilitation. ④ For patients with severe sleep disorders, the causes should be analyzed and addressed, and sleep aids should be prescribed if necessary. ⑤ Pain management: Fully assess the degree of pain and administer pain relief medications as appropriate.
Exercise training	① Low-risk patients: a. Inspiratory muscle training (IMT): Patients should be seated, inhale quickly and exhale slowly, starting with 0-level resistance, gradually increasing resistance. Perform 30 repetitions per set, 1-2 sets per day. b. Aerobic exercise training: Activities such as brisk walking, cycling, and jogging, with a modified Borg scale score of 13-16 points, lasting 30-40 minutes (including 5 minutes of warm-up, 20-30 minutes of exercise at target intensity, and 5 minutes of cool-down), 3 times per week; c. Resistance training: Performed in a seated or supine position, using resistance bands or body weight for exercises such as seated knee lifts or chest expansion. Intensity should be based on 8-15 maximum repetitions (RM), with 10 repetitions per set, 2 sets per session, twice a week. ② Moderate- to high-risk patients: After a comprehensive assessment, if applying the general plan described in ①, patients should first establish basic cardiopulmonary function. Exercises should be conducted under the guidance of a nurse, with a personalized plan implemented under supervision if necessary.

### 2.4 Outcome measures evaluation

#### 2.4.1 General patient data survey

This includes age, gender, educational level, BMI, surgical approach, time from diagnosis to surgery.

#### 2.4.2 Serum albumin and serum prealbumin indicators

The normal ranges for serum albumin and serum prealbumin are 35–55 g/L and 18–39 g/L, respectively.

#### 2.4.3 Hospital anxiety and depression scale score

The HADS is widely used to assess patients’ anxiety and depression levels. It consists of two subscales: the Anxiety Subscale (HADS-A) and the Depression Subscale (HADS-D), each containing 7 items. The total score for each subscale ranges from 0 to 21. A score of ≥8 indicates the presence of anxiety or depression, with higher scores reflecting greater severity.

#### 2.4.4 Six-minute walk distance

The Six-Minute Walk Test (6MWT) measures the distance a patient can walk briskly within six minutes (22). It is a standardized method that can quickly and effectively assess cardiopulmonary function, with the result, 6MWD, serving as a good indicator of the patient’s physical functional capacity.

#### 2.4.5 The MOS 36-item short-form health survey score

The SF-36 scale is a health survey questionnaire developed by the Health Institute in Boston, USA, based on the Medical Outcomes Study (MOS SF) created by Stewartse (23). The Chinese version of the SF-36 has been shown to have reliable validity and reliability in thoracic surgery (24).

#### 2.4.6 The postoperative hospital stay, the time to chest tube removal, the time to first ambulation, and the complications graded according to the Clavien-Dindo classification were collected for both groups of patients

Researchers usually reflect the postoperative recovery and prognosis of patients undergoing oesophageal surgery by the postoperative hospital stay, the time to chest tube removal, the time to first ambulation, and the complications graded according to the Clavien-Dindo classification, so we also selected the above indicators as outcome measures.

### 2.5 Data collection method

Two nursing postgraduates (uniformly trained and not involved in the intervention implementation) were responsible for distributing questionnaires and collecting data. Data on the HADS scores, albumin and prealbumin levels, and SF-36 scores were collected for both groups at four time points: before the intervention, one day before surgery, one week after surgery, and four weeks after surgery. Additionally, the 6MWD was measured before the intervention, one day before surgery, and four weeks after surgery. Postoperative hospital stay, chest drain removal timing, first ambulation timing, and complications were also recorded.

### 2.6 Statistical methods

Data were organized and analyzed using Excel and SPSS 26.0. Continuous variables were described using the median, standard deviation, mean, and the first and third quartiles, while categorical variables were presented as frequencies. The Mann-Whitney U test or independent sample *t*-test was employed to analyze continuous variables between the two groups. The chi-square test or Fisher’s exact test was used for categorical data analysis. For comparing normally distributed data across time points within a group, repeated measures analysis of variance (ANOVA) was performed. If Mauchly’s test of sphericity was met, the test results for within-subject effects were used; if not, the Greenhouse-Geisser correction was applied. Non-normally distributed data were analyzed using the generalized estimating equation (GEE).

## 3 Results

### 3.1 Comparison of general data between the two groups

A total of 96 patients participated in the study, with 2 patients lost to follow-up in the prehabilitation group (1 patient withdrew due to loss of contact, and 1 patient withdrew after doubting the intervention’s efficacy, assuming it would inevitably be effective). In the control group, 1 patient was lost to follow-up due to a change in contact information. Ultimately, 47 patients were included in the control group and 46 in the prehabilitation group, making a total of 93 cases.

The comparison of general data is shown in **Table 2**, and the baseline characteristics of the two groups are comparable.

**Table 2:** General information of two groups.

General information		Control group	Prehabilitation group	*χ* ^2^/t value	P Value
Gender	Male	27	25	0.091	0.763^a^
	Female	20	21		
Age	$\bar{X}$ ± S	68.28 ± 5.44	69.35 ± 5.50	-0.945	0.347^c^
Education level	Primary school or below	23	24	2.270	0.771^b^
	Middle school	13	12		
	High school/technical secondary school	6	8		
	Junior college	4	1		
	Bachelor’s degree or above	1	1		
BMI (kg/m^2^)	$\bar{X}$ ± S	20.91 ± 2.60	20.33 ± 2.88	1.034	0.304^c^
Medical insurance type	Worker with medical insurance	17	13	0.860	0.682^b^
	Medical insurance for residents	28	30		
	Self-paying	2	3		
Time from diagnosis to surgery (d)	$\bar{X}$ ± S	15.70 ± 1.83	15.93 ± 2.31	-0.725	0.470^c^
Surgical procedure	Thoracotomy	16	12	0.699	0.403^a^
	Thoracoscopy or combined laparoscopy	31	34		
Surgery duration (min)	$\bar{X}$ ± S	278.89 ± 64.84	289.24 ± 72.42	-5.379	0.591^c^
Pathological type	Squamous cell carcinoma	32	29	0.262	0.609^a^
	Adenocarcinoma	15	17		
Tumor stage	Stage I	14	13	0.739	0.691^a^
	Stage II	26	23		
	Stage III	7	10		
Neoadjuvant treatment	Yes	7	10	0.729	0.393^a^
	No	40	36		

^a^Chi-square test, ^b^Fisher’s exact test, ^c^Independent samples t-test.

### 3.2 Comparison of serum albumin and prealbumin between the two groups

The two groups were comparable before the intervention. At each time point after the intervention, the prehabilitation group had higher levels than the control group (*P* < 0.05). The albumin results showed a time effect, intervention effect, and interaction effect (*P* < 0.05). For prealbumin, there were time and intervention effects (*P* < 0.05), but no interaction effect (*P* > 0.05). This indicates a significant difference in albumin and prealbumin levels between the two groups due to differences in the rehabilitation care plans. Additionally, the nutritional indicators of the prehabilitation group remained more stable postoperatively. See **Tables 3** and **4** for details.

**Table 3:** Comparison of albumin between the two groups.

Group	Number of cases	Albumin (g/L)				F time	F between groups	F interaction
		Before intervention	1 day before surgery	1 week after surgery	4 weeks after surgery			
Control group	47	37.34 ± 3.42	38.68 ± 4.15	36.08 ± 4.08	34.15 ± 4.55	34.335	111.817	17.181
Prehabilitation group	46	36.60 ± 3.51	45.11 ± 4.34	43.24 ± 4.53	38.78 ± 4.16			
t-value		1.029	-7.303	-8.009	-5.131			
P value		0.306	<0.001	<0.001	<0.001	<0.001	<0.001	<0.001

**Table 4:** Comparison of prealbumin between two groups.

Group	Number of cases	Prealbumin (mg/L)				F time	F between groups	F interaction
		Before intervention	1 day before surgery	1 week after surgery	4 weeks after surgery			
Control group	47	271.24 ± 68.55	291.10 ± 65.78	274.78 ± 81.62	250.34 ± 54.20	7.624	13.105	0.858
Prehabilitation group	46	276.50 ± 67.91	323.68 ± 58.08	306.43 ± 69.95	277.50 ± 59.24			
t-value		-0.371	-2.531	-2.006	-2.307			
P value		0.711	0.013	0.048	0.023	<0.001	<0.001	0.463

### 3.3 Comparison of psychological states between the two groups

Before the intervention, the HADS-A and HADS-D scores of both groups were comparable (*P* > 0.05). After the intervention, the prehabilitation group had significantly lower scores at each time point compared to the control group (*P* < 0.05). GEE analysis indicated significant intervention effects, time effects, and interaction effects between the two groups (*P* < 0.05), demonstrating that the HADS scores of the two groups showed significant differences due to the variations in rehabilitation nursing plans. For details, see **Tables 5** and **6**.

**Table 5:** Comparison of HADS between two groups.

Group	Number of cases	HADS-A score (M, P_25_, P_75_)				HADS-D score (M, P_25_, P_75_)			
		Before intervention	1 day before surgery	1 week after surgery	4 weeks after surgery	Before intervention	1 day before surgery	1 week after surgery	4 weeks after surgery
Control group	47	8(7,10)	10(8,11)	7(5,10)	5(4,6)	8(7,9)	9(8,12)	7(5,9)	5(4,6)
Prehabilitation group	46	8(7,10)	5(3,6)	5.50(4,7)	4(3,5)	7(6,9)	3(3,5.25)	6(4,7)	4.5(3,5)
Z Value		-0.531	-6.472	-2.630	-2.004	-1.818	-7.217	-2.458	-2.153
P Value		0.585	<0.001	0.009	0.045	0.069	<0.001	0.014	0.031

**Table 6:** GEE analysis results of HADS for both groups.

Project	HADS-A			HADS-D		
	Wald χ^2^	df	P	Wald χ^2^	df	P
(Intercept)	2412.813	1	<0.001	3135.705	1	<0.001
Time	322.508	3	<0.001	171.76	3	<0.001
Group	43.372	1	<0.001	78.901	1	<0.001
Time * Group	45.741	3	<0.001	62.747	3	<0.001

### 3.4 Comparison of 6MWD between two groups

Before the intervention, the 6MWD of both groups was comparable (*P* > 0.05). Compared to the control group, the prehabilitation group showed higher 6MWD at all time points after the intervention (*P* < 0.05). A time effect, intervention effect, and interaction effect were observed between the two groups (*P* < 0.05), indicating significant differences in 6MWD due to the different rehabilitation care programs. Details are shown in **Table 7**.

**Table 7:** Comparison of 6MWD between two groups.

Group	Number of cases	6MWD (m)			F time	F between groups	F interaction
		Before intervention	1 day before surgery	4 weeks after surgery			
Control group	47	427.11 ± 65.99	451.28 ± 58.64	412.06 ± 53.74			
Prehabilitation group	46	433.85 ± 68.40	496.17 ± 68.58	478.22 ± 64.41	11.980	23.475	5.611
t-value		-0.484	-3.396	-5.383			
P value		0.630	0.001	<0.001	<0.001	<0.001	0.004

### 3.5 Comparison of quality of life between two groups

Before the intervention, there was no significant difference in the SF-36 scores between the two groups (*P* > 0.05). After the intervention, at each time point, the SF-36 scores of the experimental group were higher than those of the control group (*P* < 0.05). There was a time effect, intervention effect, and interaction effect between the two groups’ SF-36 scores (*P* < 0.05), as detailed in **Table 8**.

**Table 8:** Comparison of SF-36 scale scores between two groups of patients.

Project	Group	Before intervention	1 day before surgery	1 week after surgery	4 weeks after surgery	F time	F between groups	F interaction
SF-36 Score	Control group	44.11 ± 4.27	49.06 ± 3.95	43.60 ± 3.70	56.15 ± 4.64	242.165	105.643	9.128
	Prehabilitation group	45.48 ± 2.93	51.96 ± 4.78	50.13 ± 3.86	62.04 ± 2.65			
	t Value	-1.801	-3.182	-8.373	-7.497			
	P Value	0.075	0.002	<0.001	<0.001	<0.001	<0.001	<0.001

### 3.6 Comparison of postoperative hospitalization, chest drain removal, time to first ambulation, and complications between two groups

(1) The prehabilitation group had significantly shorter postoperative hospitalization, chest drain removal, and time to first ambulation compared to the control group (*P* < 0.05), as shown in **Table 9**. (2) Complications occurred in 19 cases in the control group and 11 cases in the prehabilitation group (P>0.05), as shown in **Table 10**.

**Table 9:** Comparison of postoperative hospitalization, chest drain removal, and time to first ambulation between two groups.

Group	Number of cases	Postoperative hospital stay (days)	Chest drain removal (days)	First ambulation time (days)
Control group	47	17.64 ± 3.38	10.57 ± 3.20	3.98 ± 1.31
Prehabilitation group	46	14.83 ± 3.33	8.28 ± 3.13	2.59 ± 1.22
Statistics		4.043^a^	3.489^a^	-4.712^b^
PValue		<0.001	0.001	<0.001

^a^t value, ^b^Z value.

**Table 10:** Comparison of complications between two groups.

Group	Number of cases	Complications (case)					
		Grade I	Grade II	Grade III	Grade I V	Grade V	Total
Control group	47	7	6	3	2	1	19
Prehabilitation group	46	5	3	2	1	0	11
χ^2^ Value	2.901 0.089						
*P* Value							

## 4 Discussion

### 4.1 The effect of prehabilitation programs on the nutritional status of elderly esophageal cancer patients

Studies have shown that the incidence of malnutrition in esophageal cancer patients is as high as 50% to 76% (25), and patients with a high risk of malnutrition tend to have worse prognoses, higher complication rates, and poorer outcomes. Preoperative nutritional support can promote wound healing, prevent complications, and enhance immune function.

In this study, the prehabilitation group underwent nutritional screening and assessment prior to surgery and received personalized nutritional support. After the intervention, the perioperative nutritional indicators of the prehabilitation group were superior to those of the control group, with more stable changes in the prehabilitation group, facilitating better postoperative nutritional recovery. This finding is consistent with the results of Chen et al. (26), which demonstrated that postoperative patients, with sufficient nutritional support and coordinated exercise, successfully transitioned through the catabolic phase and experienced accelerated recovery.

### 4.2 Effect of the prehabilitation program on the psychological state of elderly patients with esophageal cancer

The incidence of esophageal cancer is higher among the elderly, and these patients tend to have more complications and higher mortality rates. Emotional disorders such as depression and anxiety are common, and some patients may develop autonomic dysfunction, all of which negatively affect postoperative recovery (27). Preoperative psychological interventions aimed at improving patients’ mental state, along with encouragement and support for prehabilitation exercises and nutritional planning, can be beneficial.

In both groups, preoperative HADS-A and HADS-D scores were greater than 8, indicating that elderly esophageal cancer patients experienced anxiety and depression prior to surgery. Psychological support provided to elderly patients may influence hormone secretion through the hypothalamic-pituitary-adrenal axis, thereby enhancing immune function and promoting physical recovery. The results showed that the HADS-A and HADS-D scores in the prehabilitation group were significantly lower than those in the control group during the perioperative period (*P*<0.05), with significant time effects, intervention effects, and interaction effects (*P*<0.05). This suggests that the prehabilitation program is effective in alleviating anxiety and depression in patients. A study by Zhou X et al (28) on the impact of prehabilitation on depression scores in elderly patients undergoing laryngeal surgery also demonstrated that prehabilitation effectively alleviates depression, consistent with the findings of this study.

### 4.3 Effects of prehabilitation programs on perioperative functional status and physical capacity in elderly patients with esophageal cancer

Elderly patients with esophageal cancer often experience increased metabolism, more complications, and an elevated physical and mental burden due to treatment, which frequently leads to greater consumption of energy and a significant decline in functional capacity (29). These factors negatively impact postoperative recovery, leading to more adverse events, extended hospital stays, and delayed rehabilitation. Exercise prehabilitation can trigger physiological stress, promoting adaptive responses in tissues and organs, which enhances the patient’s ability to cope with surgical stress (29).In this study, the prehabilitation group underwent a comprehensive assessment of functional status, and personalized exercise plans were developed based on each patient’s condition, ensuring safety while improving physical function. The results showed that implementing the prehabilitation program was beneficial in increasing patients’ 6MWD during the perioperative period, as well as improving functional status and physical capacity. This is consistent with the findings of Moorthy et al. (30), where systematic prehabilitation exercise training increased patients’ preoperative functional reserve, enhancing endurance and physical fitness, and resulting in faster and better recovery compared to conventional care after surgery.

### 4.4 Effect of prehabilitation program on quality of life of elderly patients with esophageal cancer

Elderly patients with esophageal cancer are often affected by cachexia, which reduces physiological reserves. When combined with surgical trauma, this results in poor quality of life (31). In this study, the SF-36 scale was used to explore the impact of the prehabilitation program on quality of life. The results showed that the implementation of the prehabilitation program was effective in improving patients’ overall quality of life. This finding is similar to that of Allen et al. (13), and may be related to the improvement of both physiological reserves and psychological well-being as a result of prehabilitation.

### 4.5 Effect of prehabilitation program on postoperative hospitalization, chest drain removal, first ambulation, and complications in elderly patients with esophageal cancer

Patients in the prehabilitation group ambulated earlier, had their chest drains removed sooner, and experienced shorter postoperative hospital stays, consistent with the findings of Swaminathan et al. (32). This suggests that the prehabilitation program, which provides psychological, nutritional, and exercise support, contributes to improving patients’ mental health, enhancing muscle mass and mobility, and facilitating the recovery of their functional status and ability to ambulate. It also helps shorten the time needed for chest drain removal and hospital stay.

Although some studies (33)indicate that prehabilitation can reduce the incidence of postoperative complications by improving patients’ physical and psychological reserves, other research shows that prehabilitation does not effectively lower the complication rate (30), which aligns with the findings of this study. This discrepancy may be related to the complexity and difficulty of treating elderly esophageal cancer patients. Therefore, future studies could consider conducting more rigorous, large-scale, multicenter randomized controlled trials to further evaluate the effect of prehabilitation on complications in elderly patients with esophageal cancer.

### 4.6 Artificial intelligence influence on perioperative management

In our research, all indicators of the prehabilitation group were better than those of the control group, except for complications. Maybe there are other variables which were not studied such as stage of the disease the stage of training of the operating surgeon even the time when the operation was carried out first on the list or second on the list. We notice that deep learning used in AI can help identify such variables even with small sample size, deep learning using neural networks can be used instead of basic statistics (34, 35). And AI can be used in enhancing perioperative care and even intraoperative experience such as using Deepseek to answer some questions from patients about prehabilitation (36). In future research, we can attempt to explore the use of AI to manage patients who participated in prehabilitation, in order to improve the scientific and rigorous nature of prehabilitation management.

## 5 Conclusion

This study suggests that implementing a prehabilitation program for elderly esophageal cancer patients holds significant value. It can improve patients’ nutritional status, enhance physical endurance and quality of life, and to some extent, alleviate perioperative anxiety and depression, as well as shorten postoperative recovery time. However, it does not reduce the incidence of complications which maybe related to other variables, in future research, we can introduce AI to control variables and strengthen communication with patients. Compared to younger individuals, elderly patients have weaker physiological and psychological vulnerabilities, so a comprehensive monitoring of all patient indicators should be conducted throughout the prehabilitation process for safety reasons. Furthermore, as this was a single-center study with a relatively small sample size, potential biases may exist, and future large-scale multicenter trials are needed to further verify its scientific validity and feasibility.

## Data Availability

The raw data supporting the conclusions of this article will be made available by the authors, without undue reservation.
